# Guarana (*Paullinia cupana*) Stimulates Mitochondrial Biogenesis in Mice Fed High-Fat Diet

**DOI:** 10.3390/nu10020165

**Published:** 2018-01-31

**Authors:** Natália da Silva Lima, Lucimara Teixeira, Alessandra Gambero, Marcelo Lima Ribeiro

**Affiliations:** Laboratory of Immunopharmacology and Molecular Biology, Clinical Pharmacology and Gastroenterology Unit, Sao Francisco University Medical School, Av Sao Francisco de Assis, 218, Braganca Paulista-SP 12916-900, Brazil; lucimara.teixeira@usf.edu.br (L.T.); alessandra.gambero@usf.edu.br (A.G.)

**Keywords:** guarana (*Paullinia cupana* Kunth), obesity, mitochondrial biogenesis, energy expenditure

## Abstract

The aim of this study was to evaluate the effects of guarana on mitochondrial biogenesis in a high-fat diet (HFD)-fed mice. C57BL6J mice were divided in two groups: high-fat diet HFD and high-fat diet + guarana (HFD-GUA). Both groups received HFD and water ad libitum and the HFD-GUA group also received a daily gavage of guarana (1 g/kg weight). Body weight and food intake was measured weekly. Glycemic, triglyceride, and cholesterol levels were determined. VO_2_ and energy expenditure (EE) were determined by indirect calorimetry. Gene expression was evaluated by real-time PCR and protein content by western blotting. The HFD-GUA group presented lower body weight, subcutaneous, retroperitoneal, visceral, and epididyimal adipose tissue depots, and glycemic and triglyceride levels, with no change in food intake and cholesterol levels. Furthermore, the HFD-GUA group presented an increase in VO_2_ and basal energy expenditure (EE), as well as *Pgc1*α, *Creb1*, *Ampka1*, *Nrf1*, *Nrf2*, and *Sirt1* expression in the muscle and brown adipose tissue. In addition, the HFD-GUA group presented an increase in mtDNA (mitochondrial deoxyribonucleic acid) content in the muscle when compared to the HFD group. Thus, our data showed that guarana leads to an increase in energetic metabolism and stimulates mitochondrial biogenesis, contributing to control of weight gain, even when associated with high-fat diet.

## 1. Introduction

Obesity is a major public health problem worldwide and is related to epigenetic factors, excessive consumption of processed food rich in fat and sugar, and lack of physical activity, among other factors. The World Health Organization (WHO) defines overweight and obesity as abnormal or excessive fat accumulation that contribute to development of other diseases, such as diabetes mellitus, hypertension, and kidney or coronary problems [1,2].

Energy metabolism is determined by energy expenditure and food intake, which must be balanced for body weight maintenance. Skeletal muscle is a target organ in the context of cellular bioenergetics due to its important role in glucose homeostasis and insulin sensitivity [3,4]. Thus, it is common that obese subjects, that normally present an increase body fat mass and decrease in fat free mass, have a decrease in energy expenditure.

Peroxisome proliferator-activated receptor gamma coactivator 1-alpha (*Pgc1α*) is responsible for regulating mitochondrial biogenesis, oxygen consumption, and oxidative phosphorylation, through the increase of mitochondrial mass, activation of several key components of adaptive thermogenesis, and stimulation of energy uptake, which allows the adaptation of cells and tissues to situations of high energy demands [5]. *Pgc-1α* is regulated by posttranslational modification, including phosphorylation and deacetylation by protein kinase, AMP-activated (*Ampk*) and sirtuin 1 (*Sirt1*), respectively [6]. These three genes compose an energy-sensing network that controls energy expenditure in skeletal muscle [7].

Several strategies for obesity control have been developed, and functional food and/or bioactive compounds with thermogenic effects have been widely used. Green tea has been associated with weight loss and the modulation of energy expenditure and fat metabolism [8]. Consumption of curcumin, a member of the ginger family, increases thermogenic gene expression (such as uncoupling protein 1 (*Ucp1*) and *Pgc1a*) and increases mitochondrial content in inguinal white adipose tissue [9]. In addition, in C57BL6 mice fed with HFD and luteolin, a natural flavonoid abundant in pepper, celery, thyme, peppermint and honeysuckle, an increase in oxygen consumption, as well as higher carbon dioxide production and respiratory exchange ratio was observed [10]. Furthermore, resveratrol was able to reduce oxidative stress, restoring mitochondrial functional activities and stimulating oxidative phosphorylation and mitochondrial biogenesis gene expression in high-fat diet fed mice [11,12].

Guarana (*Paullinia cupana* Kunth) has been associated with weight loss, showing several protective actions against hypertension, obesity, and metabolic syndrome [13], the capacity to reduce food intake [14], and to modulate genes related to adipogenesis [15]. Besides that, it has already been demonstrated that a mixture of guarana extract and green tea containing a fixed dose of caffeine (200 mg) and variable doses of epigallocatechin-3-gallate (EGCG) increased energy expenditure (measured in a metabolic chamber to measure 24 h energy expenditure) in healthy adults [16]. Thus, the aim of our study was to investigate the effects and the potential mechanisms underlying the effects of oral treatment with guarana on obesity, metabolism, and mitochondrial biogenesis.

## 2. Material and Methods

### 2.1. Experimental Design

All animal experiments were performed in accordance with the Brazilian Government’s ethics and were approved by the Animal Experimental Ethics Committee (CEUA) of the Sao Francisco University, Bragança Paulista, SP, Brazil, under protocol no. 001.05.2015. All procedures including housing and welfare were carried out in accordance with the recommendations in the Guiding Principles for Biomedical Research Involving Animals of the International Council for Laboratory Animal Science (ICLAS), countersigned by the Conselho Nacional de Controle de Experimentação Animal (CONCEA: Brazilian National Consul for the Control of Animal Experimentation). C57BL6J male mice at approximately 4 weeks of age were obtained from Sao Paulo University (Sao Paulo, Brazil) and maintained in a 12/12 h artificial light/dark cycle and temperature (22 ± 2 °C) in individual cages for 4 weeks. After randomization, C57BL6J mice were given either a high-fat diet (HFD group, *n* = 6) or a high-fat diet + guarana (*Paullinia cupana*) (1 g/kg of body weight) (HFD-GUA group, *n* = 6) for 8 weeks. Guarana was administered daily by gavage using an orograstric cannula. HFD group received gavage with pure water (vehicle) in the similar volume of HFD-GUA group for 8 weeks. The composition of the experimental diet is presented in Table 1. In addition, the composition of guarana used in this study was previously determined; 2.42% of flavonoids, 9.18% of total phenolics, and high caffeine content (12.4%) [15].

### 2.2. Indirect Calorimetry

Basal energy expenditure of the animals was evaluated by indirect calorimetry. Forty-eight hours before euthanasia, mice were acclimatized for 24 h in individual metabolic cages (OXYLET System—for rodents) and monitored for another 24 h. The amount of O_2_ (VO_2_) consumed and the amount of CO_2_ (VCO_2_) produced was measured at 25-min intervals for 24 h. Respiratory exchange rate (RER) was calculated using the following formula: (RER) = VCO_2_/VO_2_. Basal energy expenditure (EE) was determined using following formula: kcal/day/kg ^0.75^ = 1.44 × VO_2_ × (3815 + 1232 × RER) [17].

### 2.3. Animal Procedure and Tissue Dissection

Food intake and body weight was measured weekly. Glycemic, triglycerides, and cholesterol levels were determined by Accutrend Plus (Roche Diagnostics GmbH, Mannheim, Germany) using specific strips. At the end of 8 weeks, mice were anesthetized (after 12 h of fasting) with a 1:1 solution of 2% xylazine/10% ketamine (1 μL/g of body weight) and blood samples were collected by cardiac puncture. Adipose tissue depots (subcutaneous, retroperitoneal, visceral, and epididyimal) were dissected and weighed. Gastrocnemius muscle and brown adipose tissue samples were dissected and stored at −80 °C until analysis.

### 2.4. mRNA (Messenger Robonucleic Acid) Expression Analysis

Muscle samples were used for quantitative real time PCR analysis. Total RNA extraction, complementary DNA (cDNA) synthesis, and quantitative PCR were performed as previously described [18], using specific primers (Table 2). First, all samples were normalized using housekeeping *18S* gene and after, the HFD-GUA group was normalized by HFD group. The equation 2^−ΔΔ*C*t^ was used to calculate the fold change.

### 2.5. Mitochondrial DNA Quantification (Mtdna)

Mitochondrial DNA (mtDNA) quantification was performed by quantitative real time-PCR. Briefly, DNA (deoxyribonucleic acid) extraction (from muscle and brown adipose tissue) was performed using the phenol/chloroform method. Next, real time-PCR was performed using Platinum^®^ SYBR GREEN^®^ qPCR Supermix Uracil-DNA-glycosylase (UDG) (Invitrogen, CA, USA) according to the manufacturer’s protocol. For mtDNA quantification, we used mitochondrial *Cox1* (cytochrome c oxidase subunit I) (FW 5′-GCCCCAGATATAGCATTCCC-3′ and RV 5′-GTTCATCCTGTTCCTGCTCC-3′) and as an endogenous control, *18S* rRNA (FW 5′-TAGAGGGACAAGTGGCGTTC-3′ and RV 5′-CGCTGAGCCAGTCAGTGT-3′). Real time-PCR was performed in a 7500 real-time PCR system (Applied Biosystems Foster City, CA, USA) and analyzed using RQ Study Software (Applied Biosystems). The relative quantification of mtDNA copies was obtained by the DNAmt/nuclear DNA ratio, and after normalization with housekeeping gene *18S*, the fold change was determined using the equation: 2^−ΔΔ*C*t^ method.

### 2.6. Western Blotting Analysis

To obtain cell extracts, samples of muscle and brown adipose tissue were treated to protein extraction using ice-lysis buffer containing protease inhibitors. Subsequently, the homogenates were centrifuged (12,000 rpm, 4 °C, 10 min) and stored at −80 °C. Briefly, protein concentrations were determined by the bicinchoninic acid protein assay kit (Thermo Scientific, Rockford, IL, USA). Samples (30 μg of total protein) were separated by 8.5% SDS-PAGE (sodium dodecyl sulfate polyacrylamide gel electrophoresis) according to the molecular weight of each protein and transferred to nitrocellulose membranes (Hybond ECL; Amersham Pharmacia Biotech, Amersham, London, UK). Rainbow standard markers (Kaleidoscope^TM^, Bio-Rad Laboratories, Hercules, CA, USA) were run in parallel to estimate molecular weights. The membranes were blocked with 5% blotting-grade blocker (Bio-Rad Laboratories) in Tween-Tris-buffered saline (20 mM-Tris-HCl, pH 7.5, 500 mM-NaCl and 0.1% Tween-20) for 1 h. Specific primary antibodies were used, anti-*Aampk*, anti-*p-Ampk*, anti-*Pgc1α*, anti-*Ucp1*, anti-*Ucp3*, anti- Oxidative phosphorylation (OXPHOS) (complexes I to V), and anti-vinculin (Abcam, Cambridge, UK) for 1 h. Subsequently, samples were incubated in specific HRP-linked secondary antibodies (DAKO Corporation, Hamburg, Germany) for more 1 h. The targeted proteins were detected by enhanced chemiluminescence (Amersham Pharmacia Biotech, Piscataway, NJ, USA) and then exposed to X-ray film. The images were scanned, and the bands were quantified by densitometry using Image J 1.34 s software (Wayne Rasband National Institute of Health, Bethesda, MA, USA). All measurements were normalized to the vinculin protein band intensity.

### 2.7. Statistical Analysis

Data are presented as mean values ± SEM. GraphPad Prism 5 was used for statistical analyses and graphics (GraphPad Software, Inc., San Diego, CA, USA). Experimental data were analyzed by Student’s unpaired t test. For body weight analysis, two-Way Analysis of Variance (ANOVA) was used.

## 3. Results and Discussion

The effects of guarana (*Paullinia cupana*) extract on body weight control, food intake, protection against hypertension, and in the modulation of some genes and miRNAs associated with the adipogenesis process have been previously shown [13,14,15]. In addition, in this study, it was demonstrated that guarana might control body weight by enhancing thermogenesis and mitochondrial biogenesis.

**Figure 1:**
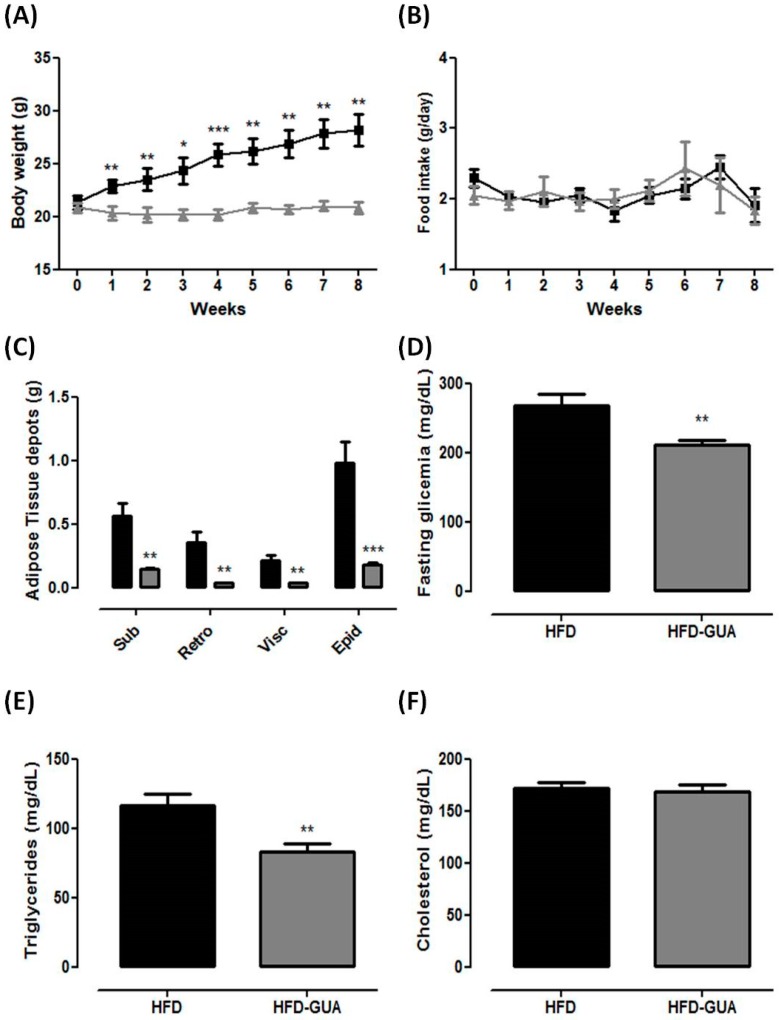
(**A**) Body weight (g) of high fat diet (HFD)-group (*n* = 6) and high fat diet + Guarana (HFD-GUA) group (*n* = 6) during eight weeks of treatment; (**B**) Food intake (g) of HFD-group (*n* = 6) and HFD-GUA group (*n* = 6) during eight weeks of treatment; (**C**) Adipose tissue depots (g) of Sub—subcutaneous adipose tisse, Retro—retroperitoneal adipose tissue, Visc—visceral adipose tissue and Epi—epididyimal adipose tissue after eight weeks of treatment; (**D**) Glycemia (mg/dL) after 12 h of fasting; (**E**) Triglycerides (mg/dL) after 12 h of fasting; (**F**) Cholesterol (mg/dL) after 12 h of fasting. Black line/bars correspond to HFD-group (*n* = 6) and grey line/bars correspond to HFD-GUA group (*n* = 6). * *p* < 0.05, ** *p* < 0.01 and *** *p* < 0.001 when compared with HFD group.

Our data show that the HFD group presented an increase in body weight from the first week, while the HFD-GUA group did not change in body weight for the entire period of experiment (Figure 1A). These data confirm that guarana is able to prevent weight gain (even associated with HFD), despite the same food intake for both groups in this experimental model (Figure 1B). These results indicate that the consumption of guarana, even when associated with an increased calorific intake, was able to prevent weight gain independently of satiety, since the food intake was not changed during the experiment. In obese subjects, however, a formulation containing guarana with yerba mate and damiana (denominated “YGD”) was associated with a decrease in food intake [14]. In a recent randomized, single blind, placebo-controlled study realized with overweight and obese women, YGD was able to modulate gastrointestinal hormones, leading to a decrease in acylated ghrelin and an increase in glucagon-like peptide 1 (GLP-1) levels that caused a reduction of energy and macronutrient intake [19]. Additionally, guarana also showed protective effect against hypertension, obesity, and metabolic syndrome in elderly healthy volunteers [13]. Recently, it has been shown that the consumption of a multi-ingredient product (containing guarana in addition to many other compounds) in healthy subjects lead to an increase in fatty acid oxidation, decrease in rate of perceived exertion during exercise, and improved satiety [20].

Furthermore, HFD-GUA mice showed a decrease in subcutaneous, retroperitoneal, visceral and epididyimal adipose tissues weight (−74%, −90%, −83% and −82%, respectively) when compared to mice fed with HFD. These data indicate that guarana is able to prevent adipose tissue accumulation. Fasting glycemic and triglyceride levels were lower in the HFD-GUA group when compared to those in the HFD group (−21% and −28%, respectively), with no difference in total cholesterol. Accordingly, previous data from our group indicated that guarana modulates adipogenesis [15] as well as increases fatty acid oxidation [20], which could contribute to the observed results here.

**Figure 2:**
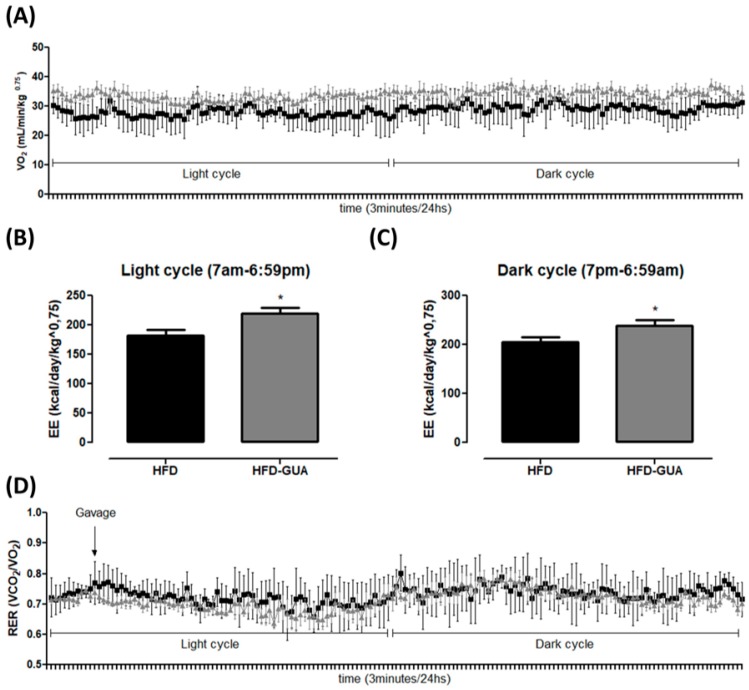

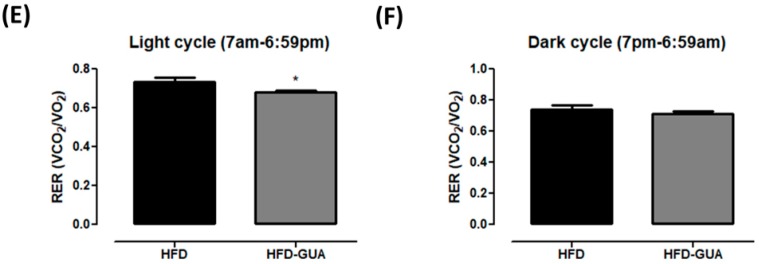
(**A**) Illustrative evolution of VO_2_ (mL/min/kg^0.75^) during 24 h after eight weeks of treatment. Light cycle (7 a.m. to 6:59 p.m.) and dark cycle (7 p.m. to 6:59 a.m.); (**B**) Energy expenditure (EE) (kcal/day/kg^0.75^) in the light cycle after weigh weeks of treatment; (**C**) EE (kcal/day/kg^0.75^) in the dark cycle after weigh weeks of treatment; (**D**) Illustrative evolution of Respiratory exchange ratio (RER) during 24 h after eight weeks of treatment; (**E**) Respiratory exchange ratio (RER) in the light cycle; (**F**) Respiratory exchange ratio (RER) in the dark cycle. Black line/bars correspond to HFD-group (*n* = 6) and grey line/bars correspond to HFD-GUA group (*n* = 6). * *p* < 0.05.

Additionally, the data presented indicate that guarana can exert some effects on energy expenditure and metabolism. Indirect calorimetry showed that guarana treatment was able to increase VO_2_ in light cycle to a greater extent than in the dark cycle (Figure 2A), and also EE (+20% in the light cycle and +16% in the dark cycle, *p* < 0.05, Figure 2B,C) when compared to the HFD group. Despite mice presenting night activity, it is possible that the major increase in EE in the light cycle (+20%) is due to the gavage being performed during this cycle and not in the dark cycle. Furthermore, respiratory exchange ratio (RER) was calculated to determine if predominant fuel source was carbohydrate or fat. It is known that RER of 0.70 indicates predominant fat oxidation; RER of 0.85 suggests a mix of fat and carbohydrates, and a value of 1.00 or above is suggestive of carbohydrate oxidation [21,22]. Our data showed a decrease of RER in the light cycle mainly after gavage (Figure 2D) in the HFD-GUA group when compared to the HFD group (Figure 2E), which indicates increased fatty acid utilization in animals treated with guarana. However, we do not observe a difference in RER in the dark cycle (Figure 2F). Accordingly, guarana extract consumed together with green tea and EGCG was able to increase energy expenditure (measured in a metabolic chamber to measure 24 h energy expenditure) in healthy adults [16]. It is known that guarana has a high concentration of caffeine [15,23], and some studies have already demonstrated that caffeine is able to modulate metabolism and energy expenditure [24,25,26].

Caffeine is a natural ingredient in tea and coffee that demonstrates several beneficial actions in weight loss and other anti-obesity effects without undesirable effects [27,28]. Recently, it was demonstrated that caffeine can regulate energy metabolism through the modulation of hypothalamic neuronal activities. Peripheral administration of caffeine (by gavage) in diet-induced obese (DIO) mice reduced their food intake, increased wheel running activities, consumption of O_2_, and CO_2_ production [26]. Other recent study demonstrated that C57BL6 mice that received caffeine showed a lipolysis improve which resulted in considerable weight loss [29]. Furthermore, the administration of caffeine to the mouse brain increased the number of c-Fos^+^ cells in regions of the hypothalamus, such as the paraventricular nucleus (PVN), arcuate (Arc), and ventromedial and dorsomedial (DMH) nuclei. These results indicate that caffeine stimulates neuron activity involved with energy balance control [26]. In humans, a randomized, double-blind, placebo-controlled and cross-over trial with healthy female volunteers showed that the consumption of a thermogenic supplement (containing compounds rich in caffeine) was able to increase resting metabolic rate at 60 min, 120 min, and 180 min post ingestion compared to baseline values [30]. In vitro experiments showed that caffeine (250 and 500 μM) was able to increase relative metabolic rate in human muscle rhabdomyosarcoma cells after 24 h of incubation [25]. These results are in agreement with our findings and it is plausible that the high caffeine content in guarana extract used [15] may have contributed to increase in VO_2_ and EE in these mice.

**Figure 3:**
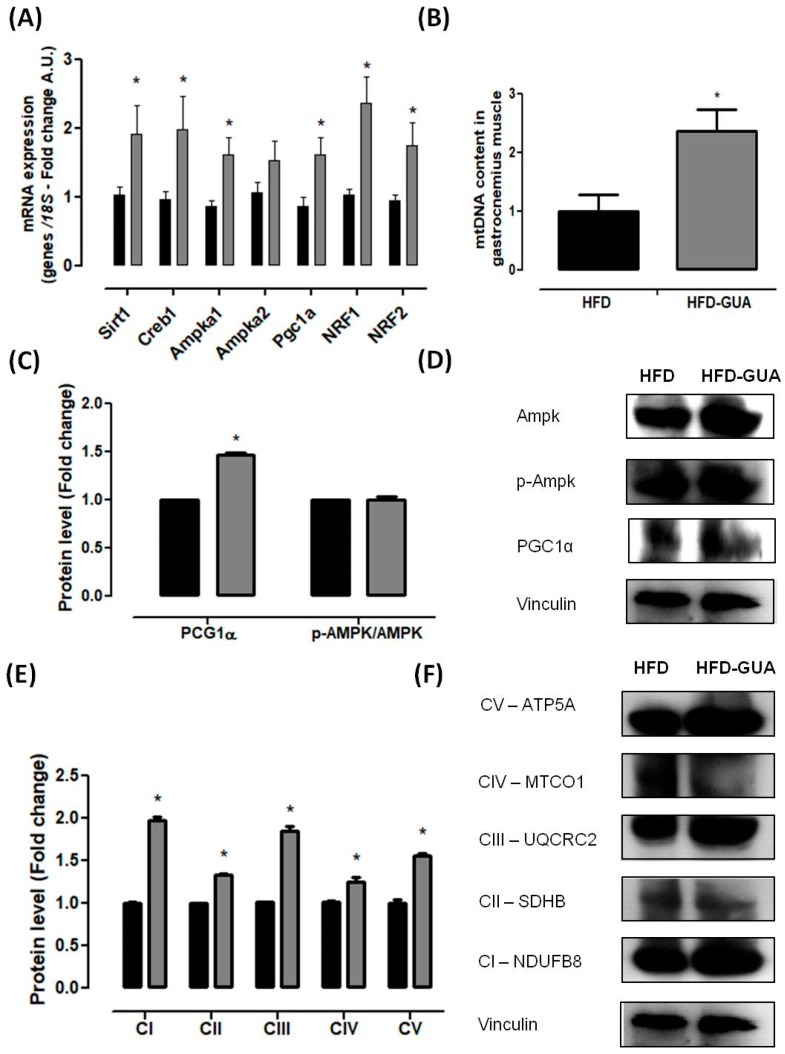
(**A**) Muscle mRNA expression of *Pgc1α*, *Creb1*, *Ampka1*, *Ampka2*, *Mkp1*, *Slc2a4*, *Nrf1*, *Nrf2*, *Sirt1* and *Mttfa* after eight weeks of treatment; (**B**) mtDNA quantification after eight weeks of treatment in gastrocnemius muscle; (**C**,**D**) Muscle protein level of *PGC1α* and *p-Ampk/Ampk* and illustrative images of protein content by western blotting; (**E**,**F**) Muscle protein level of subunits of the five OXPHOS complexes and illustrative images of protein content by western blotting. All samples were normalized using housekeeping *18S* gene and, afterwards, the HFD-GUA group was normalized by the HFD group. The equation 2^−ΔΔ*C*t^ was used to calculate the fold change. * *p* < 0.05 using Student’s *t*-test. Black bars correspond to the HFD group (*n* = 6) and grey bars correspond to the HFD-GUA group (*n* = 6). Error bars reflect SEM.

**Figure 4:**
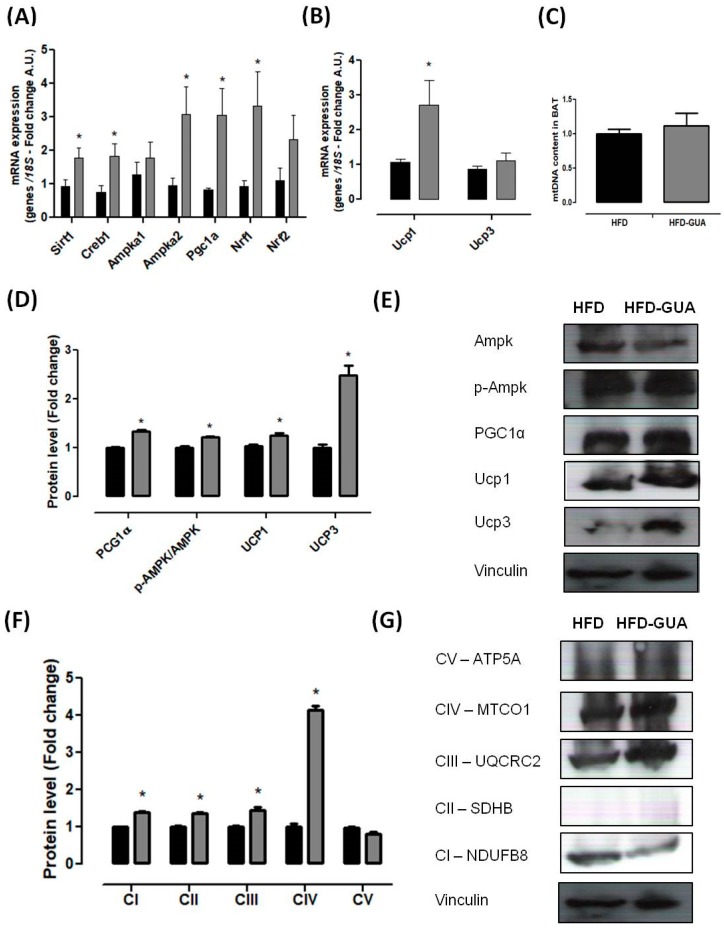
(**A**) Brown adipose tissue mRNA expression of *Pgc1α*, *Creb1*, *Ampka1*, *Ampka2*, *Nrf1*, *Nrf2* and *Sirt1* after eight weeks of treatment; (**B**) Brown adipose tissue mRNA expression of *Ucp1* and *Ucp3* after eight weeks of treatment; (**C**) mtDNA quantification after eight weeks of treatment; (**D**,**E**) Brown adipose tissue protein level of *Pgc1α*, *p-Ampk/Ampk*, *Ucp1* and *Ucp3*; and illustrative images of protein content by western blotting; (**F**,**G**) Brown adipose tissue protein level of subunits of the five OXPHOS complexes and illustrative images of protein content by western blotting. All samples were normalized using housekeeping *18S* gene and, afterwards, the HFD-GUA group was normalized by the HFD group. The equation 2^−ΔΔ*C*t^ was used to calculate the fold change. * p < 0.05 using Student’s *t*-test. Black bars correspond to the HFD-group (*n* = 6) and grey bars correspond to the HFD-GUA group (*n* = 6). Error bars reflect SEM.

**Figure 5:**
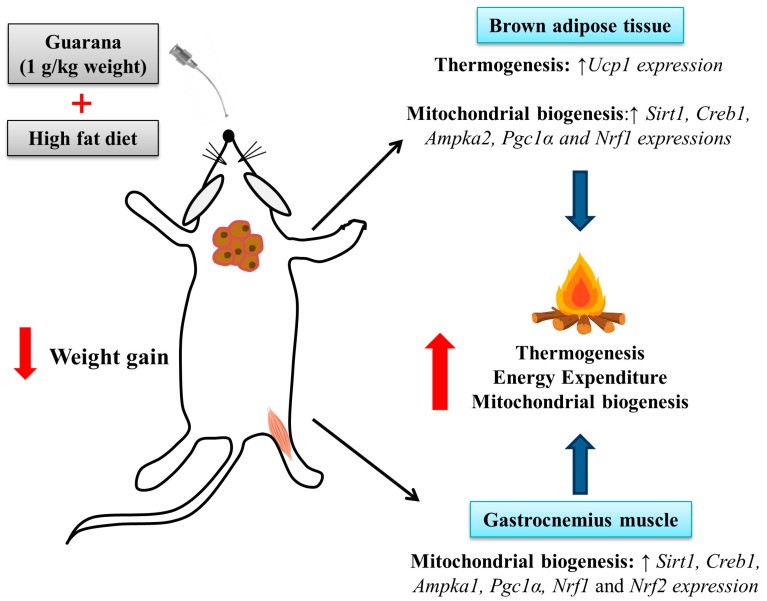
Effects of guarana treatment on mitochondrial biogenesis in gastrocnemius muscle and brown adipose tissue.

Mitochondria play a key role in energy metabolism in many tissues such as skeletal and cardiac muscle, as well as in the liver, brain, and adipose tissue [31]. Our data shows that treatment with guarana for eight weeks increased the amount of mtDNA in the gastrocnemius muscle (Figure 3B), but this difference was not observed in the brown adipose tissue (Figure 4C). Complementarily, gastrocnemius muscle of the HFD-GUA group showed up-regulated expression of mitochondrial biogenesis genes, such as *Sirt1*, *Creb1*, *Ampka1*, *Pgc1α*, *Nrf1*, and *Nrf2* (Figure 3A). With regard to brown adipose tissue, our data showed that animals treated with guarana (HFD-GUA group) showed up-regulated expression of mitochondrial biogenesis genes, such as *Sirt1*, *Creb1*, *Ampka2*, *Pgc1α*, and *Nrf1* (Figure 4A) and of thermogenic genes, such as *Ucp1* (Figure 4B) when compared to the HFD group. These data probably contributed to the increase in energy expenditure of the HFD-GUA group as compared to HFD group. The results obtained by western blotting showed the same characteristics, such as higher *Pgc1-α* in the gastrocnemius muscle (Figure 3C,D) as well as a higher content of *p-Ampk/Ampk*, *Pgc1-α*, and *Ucp1* in the brown adipose tissue (Figure 4D,E). Furthermore, the data showed an increase of OXPHOS complex I, II, III, IV and V in the gastrocnemius muscle (Figure 3E,F) and an increase of OXPHOS complex I, II, III and IV in the brown adipose tissue (Figure 4F,G). These data demonstrated that mitochondrial biogenesis and thermogenesis induced by guarana is not restricted to skeletal muscle, since the modulation was also observed in the brown adipose tissue. Other authors have already demonstrated that functional food and its bioactive compounds can modulate the expression of mitochondrial biogenesis-related genes. Resveratrol (mixed with a pelleted high-fat diet) was able to up-regulate uncoupling protein 2 (*Ucp*2) and nuclear respiratory factor 1 and 2 (*Nrf1* and *Nrf2*) expression as compared to mice fed with a high-fat diet without resveratrol [32]. Caffeine induces increases in the mRNA levels and protein levels of a number of mitochondrial enzymes in rat epitrochlearis muscle after 18 h of incubation [33], as well as being able to modulate hypothalamic neuronal activities, increasing energy expenditure and thermogenesis [26]. Taken all together, the modulation caused by guarana treatment contributed to the lower fat accumulation in all adipose tissue depots and the increase in energy expenditure, as well as the higher content of mitochondria in skeletal muscle. This action is demonstrated in Figure 5.

## 4. Conclusions

This study demonstrated that guarana prevents adipose tissue accumulation, as well as increasing energy expenditure, probably because of the modulation of several genes related to mitochondrial biogenesis in gastrocnemius muscle and brown adipose tissue. Animals treated with guarana, even when fed with a high-fat diet, presented lower body weight, lower fat deposits, and higher energy expenditure. Together, our data demonstrate that guarana presents an important role in metabolism and energy expenditure that could contribute to the therapeutic treatment of obesity.

## Figures and Tables

**Table 1:** High-fat diet composition.

Composition	High-Fat Diet	
	g kg^−1^	kcal kg^−1^
**Cornstarch**	115.5	462
**Casein**	200	800
**Sucrose**	100	400
**Dextrinated starch**	132	528
**Lard**	312	2808
**Soybean oil**	40	360
**Cellulose**	50	-
**Mineral mix**	35	-
**Vitamin mix**	10	-
**l-cystine**	3	-
**Choline**	2.5	-
**TOTAL**	1000	5358

**Table 2:** Primers used for real-time PCR.

Gene	Primer	Sequence (5′→3′)
***Sirt1***	Sense	AGTGGCACATGCCAGAGTC
	Antisense	TCCAGATCCTCCAGCACAAT
***Creb1***	Sense	TTTGTCCTTGCTTTCCGAAT
	Antisense	CACTTTGGCTGGACATCTTG
***Ampka1***	Sense	TGAGAACGTCCTGCTTGAATG
	Antisense	ATCATTGGCTGAGCCACAGC
***Ampka2***	Sense	ACAGGCCATAAAGTGGCAGT
	Antisense	GTCGGAGTGCTGATCACGTG
***Pgc1α***	Sense	CCGAGAATTCATGGAGCAAT
	Antisense	TTTCTGTGGGTTTGGTGTGA
***Nrf1***	Sense	CAACAGGGAAGAAACGGAAA
	Antisense	CACTCGCGTCGTGTACTCAT
***Nrf2***	Sense	AGGACATGGAGCAAGTTTGG
	Antisense	TCTGTCAGTGTGGCTTCTGG
***Ucp1***	Sense	TCAGGGCTGAGTCCTTTTGT
	Antisense	CTGAAACTCCGGCTGAGAAG
***Ucp3***	Sense	CTCACTTTTCCCCTGGACAC
	Antisense	GTCAGGATGGTACCCAGCAC
***18S***	Sense	AAACGGCTACCACATCCAAG
	Antisense	CAATTACAGGGCCTCGAAAG
